# Editorial: Rising stars in aging, metabolism and redox biology

**DOI:** 10.3389/fragi.2023.1218676

**Published:** 2023-05-26

**Authors:** Xiaoyong Yang, Jianhua Zhang

**Affiliations:** ^1^ Department of Comparative Medicine, Yale University School of Medicine, New Haven, CT, United States; ^2^ Department of Cellular and Molecular Physiology, Yale University School of Medicine, New Haven, CT, United States; ^3^ Yale Center for Molecular and Systems Metabolism, Yale University School of Medicine, New Haven, CT, United States; ^4^ Department of Pathology, University of Alabama-Birmingham, Birmingham, AL, United States

**Keywords:** klotho, diabetes, heart, brain, red blood cells, microbiome, lysosomes, iron

In this editorial, we highlight Rising Stars in Aging, Metabolism and Redox Biology. The role of Klotho in age-related pathologies and response to anti-diabetic drugs, cardiac dysfunction caused by diabetes and mechanisms involving the lysosomes, iron metabolism in the heart and the brain, as well as lifestyle modifications and related changes of oxidative stress markers, have been reviewed by early stage investigators. This Research Topic also includes a brief research report that evaluated the relationship of antioxidants with red blood cell storage, and a research article that reported oral and fecal microbiomes in human aging.

In this Rising Stars in Aging, Metabolism and Redox Biology Research Topic, we celebrate work performed by early stage investigators in the aging field.

Dr. Gérald Prud’homme (University of Toronto, Canada), and and Dr. Qinghua Wang (Huashan Hospital and Shanghai Medical School, China) collaborate on studies of autoimmune (type 1) diabetes, and regenerative therapy as applied to that disease. In recent years, their teams and others have examined the role of Klotho in protecting human beta cells, and stimulating their proliferation and regeneration. For example, in preclinical models, they observed that anti-diabetic drugs increased Klotho levels in the plasma, kidneys and pancreatic islets. Klotho protein therapy reduced beta-cell loss and increased cell numbers, whereas Klotho blockade was detrimental. Furthermore, Klotho exerted potent anti-inflammatory and anti-aging activities. In this Research Topic, Dr. Prud’homme and colleagues review many facets of Klotho biology, with a detailed discussion on the mode of action and therapeutic application of Klotho in endocrine, inflammatory and age-related pathologies (Prud’homme et al.).

Aging is associated with increased diabetes and metabolic disorders, which is a risk factor for the development of cardiovascular diseases. On the other hand, metabolic disorders can accelerate the aging process. Therefore, therapeutic measures against these conditions may slow or prevent various heart diseases, contributing to healthy aging. In a minireview article (Kobayashi et al.), Dr. Kobayashi, an assistant professor at the NYIT College of Osteopathic Medicine, and his team provide a unique perspective and insight into cardiac dysfunction caused by diabetes. They have investigated the potential impairment of cardiac function due to the damage of lysosomes, small organelles within the myocardium that play a crucial role in maintaining metabolic homeostasis. Specifically, they have focused on the lysosomal proteases that are released into the cytosol due to the permeabilization of lysosomal membranes. Through the development of therapeutic strategies targeting the maintenance of lysosomal integrity in the diabetic heart, Dr. Kobayashi is expected to make significant contributions to the field of diabetic complications and age-related cardiovascular diseases.

Shaina Rosenblum is a PhD candidate in Dr. Daniel Kosman’s group at the University at Buffalo. Their group concentrates on iron metabolism at the endothelial barriers of the heart and the brain, with a focus on mechanisms of iron trafficking across these barriers. As the lead on this project, she studies how inflammatory signals alter iron transporter localization at cell membranes and subsequent iron trafficking across the barrier endothelial cells. In her review article (Rosenblum), she highlights mechanisms of iron metabolism in the cardiovascular system. She also discusses inflammatory and iron metabolism-related aspects of cardiovascular disease, and the connections between inflammation and iron metabolism in this system. Increased inflammation with age correlates with cardiac disease. Understanding how inflammation relates to iron handling in the cardiovascular system may help to mitigate the pathologies of aging.

In a review article, Sarah Husain, a doctorate student and her colleagues searched PubMed and Web of Science for studies published in English from 2012 to 2021, that were related to lifestyle interventions on oxidative stress biomarkers in chronic diseases (Husain et al.). The authors surveyed 671 articles, with inclusion criteria related to cardiovascular diseases, type 2 diabetes mellitus, obesity, Alzheimer’s disease, Parkinson’s disease, metabolic syndrome or cancer, and exclusion criteria including age that are < 18 years, surgical intervention, and drug studies. They identified 9 articles that met the inclusion and exclusion criteria. It is noticeable that not many studies focus on relating oxidative stress markers and chronic diseases in these 10 years. Lifestyle modifications that focus on diet and physical health were shown to increase superoxide dismutase and catalase levels and decrease malondialdehyde levels. The heterogeneity of the methods and the biomarkers used in different studies highlight the importance of more comprehensive studies to help draw conclusions and to understand the timing, duration and specificity of lifestyle intervention studies and the relationships between lifestyle interventions and oxidative stress markers.

In a brief research report article, Dr. Tzounakas and colleagues provided analytical data from their early study on protective role of uric acid (UA) and ascorbic acid (AA) on stored red blood cells. In particular, they analyzed the inter-relationships of antioxidants and outcomes of red blood cell storage (Anastasiadi et al.). A paired correlation analysis of hemolysis, lipid peroxidation and reactive oxygen species was performed with samples from early, middle or late storage duration, in the presence of UA and/or AA supplements. The main findings include that hemolysis is more a function of donor features than of additive solution modifications. The levels of S-adenosyl methionine or cystathionine in early storage (day 7) cells are inversely correlated to oxidative stress of those in middle storage (day 21 or 42). Trypsin-like proteasomal activity in early storage (day 7) cells are inversely correlated to oxidative stress of late storage (day 42). These analyses provide new insights into strategies to optimize red blood cell storage.

Last but not the least, the postdoctoral researcher Dr. Zhou and colleague reported both oral and fecal microbiomes in the context of human aging (Zhou et al.). It was reported that the saliva microbiome diversity is lower and stool microbiome diversity is higher in the 13 healthy older group (mean = 77 years old) compared to the 10 young group (mean = 25 years old). However, the dominant bacterial microbiome is similar between young and old groups. The overall dissimilarity of the microbiomes is higher with stool than with saliva between the young and old groups. Statistically more abundant operational taxonomic units (OTUs) have been identified from both young and old groups for both saliva and stool samples. This is one of the first studies that simultaneously analyzed saliva and stool microbiome in young and healthy old people. This original research will warrant future studies to understand contributing factors to microbiome changes with age and age-related diseases, and inspire new approaches for promoting healthy aging and mitigating age-related diseases.

We hope that this Research Topic provides new insights into several interesting aspects of aging research and recognizes contributions to the field by early stage aging researchers.

